# Local microwave ablation with continued EGFR tyrosine kinase inhibitor as a treatment strategy in advanced non-small cell lung cancers that developed extra-central nervous system oligoprogressive disease during EGFR tyrosine kinase inhibitor treatment: A pilot study

**DOI:** 10.1097/MD.0000000000003998

**Published:** 2016-06-24

**Authors:** Yang Ni, Jingwang Bi, Xin Ye, Weijun Fan, Guohua Yu, Xia Yang, Guanghui Huang, Wenhong Li, Jiao Wang, Xiaoying Han, Xiang Ni, Zhigang Wei, Mingyong Han, Aimin Zheng, Min Meng, Guoliang Xue, Liang Zhang, Chao Wan

**Affiliations:** a Department of Oncology, Shandong Provincial Hospital Affiliated to Shandong University; b Department of Oncology, Jinan Military General Hospital of Chinese People's Liberation Army; c Imaging and Interventional Center, Sun Yat-sen University Cancer Center; d Department of Oncology, Weifang People's Hospital Affiliated to Weifang Medical University, China.

**Keywords:** acquired resistance, EGFR-TKI, microwave ablation, non-small cell lung cancer, oligoprogressive disease

## Abstract

The non-small cell lung cancer (NSCLC) patients that experienced good clinical response to epidermal growth factor receptor-tyrosine kinase inhibitor (EGFR-TKIs) will ultimately develop acquired resistance. This retrospective study was performed to explore the potential survival benefit of microwave ablation (MWA) therapy in epidermal growth factor receptor (EGFR)-mutant NSCLC that developed extra-central nervous system (CNS) oligoprogressive disease during TKI treatment.

We retrospectively analyzed 54 NSCLC patients with EGFR mutations who showed a clinical benefit from initial EGFR-TKI therapy and developed extra-CNS oligoprogressive disease at our institutions. Twenty eight patients received MWA as a local therapy for the metastatic sites and continued on the same TKIs (MWA group). The following 26 patients received systemic chemotherapy after progression (chemotherapy group). The progression-free survival (PFS1) was calculated from initiation of targeted therapy to first progression. Progression-free survival (PFS2) was defined from first progression to second progression after MWA or chemotherapy. Overall survival (OS) was calculated from the time of diagnosis to the date of last follow-up or death.

The median PFS1 for both groups was similar (median 12.6 vs. 12.9 months, HR 0.63). However, the MWA group patients had a significantly longer PFS2 (median 8.8 vs. 5.8 months, hazards ratio [HR] 0.357) and better OS (median 27.7 vs. 20.0, HR 0.238) in comparison with chemotherapy group. Multivariate analysis and the internal validation identified MWA as the main favorable prognostic factor for PFS2 and OS. In the MWA group, the median PFS2 for complete ablation was significantly longer than that for incomplete ablation (11 vs. 4.2 months, HR 0.29, *P* < 0.05).

MWA with continued EGFR inhibition might be associated with favorable progression-free survival (PFS) and OS in patients with extra-CNS oligometastatic disease. MWA as a local therapy for extra-CNS oligometastatic disease should be considered for NSCLC with acquired resistance to EGFR-TKIs.

## Abstract 2

Supplemental Digital Content is available in the text

## 1 Introduction

Activating epidermal growth factor receptor (EGFR) mutations occur in 10 to 20% of patients with non-small cell lung cancer (NSCLC) in North American and European populations and in up to 60% among Asians populations.^[1]^ Treatment of EGFR-mutant NSCLC with specific tyrosine kinase inhibitor (TKI) that target EGFR, such as gefitinib, erlotinib or afatinib, has led to remarkable tumor shrinkage and improvement in progression-free survival (PFS) and quality of life compared with standard chemotherapy.^[2–4]^ Based on the results of numerous studies, EGFR sensitizing mutant advanced NSCLC patients should receive first-line epidermal growth factor receptor-tyrosine kinase inhibitor (EGFR-TKIs) treatment.^[3,5–7]^

Despite the initial advancement of these agents, most patients ultimately develop acquired resistance to such TKIs after 1 to 2 years.^[3,4,8,9]^ Several resistant mechanisms have been identified, such as T790M missense mutation, amplification of MET, activation of alternative pathways (IGF-1, HGF, PI3CA, AXL), and, in rare instances, transformation to small-cell histology.^[10–16]^ Although studies of second-generation, irreversible EGFR-TKIs and other novel agents are ongoing, there are no currently approved targeted therapies specific for treatment of such patients upon progression. Cytotoxic chemotherapy alone used to be the standard therapeutic option at the time of progression while continuation of TKI therapy by itself or in combination with chemotherapy seems to provide continued clinical benefit.^[17–19]^ It may be in accordance with the tumor heterogeneity in the development of resistance to targeted therapies and minor tumor cell populations will still be sensitive to the previous EGFR-TKIs.

Different patterns of progressive disease may represent different biological molecular mechanisms. So, it is important to distinguish among these patterns as different therapeutic strategies may apply. Considering the growth rate and number of growing tumour lesions, progressive disease during TKI treatment can be generally distinguished in 3 patterns: intracranial disease progression, development of 1 or few distant metastatic sites while the patient remains asymptomatic, and systematic and/or symptomatic disease progression.^[20,21]^ The first two fall into the general term of oligoprogressive disease. Typically, the definition of oligoprogressive disease refers to the presence of fewer than 5 discrete metastatic sites. Local therapy with continued EGFR inhibition has been shown to be effective for treating patients with oligoprogressive disease and associated with long PFS and OS.^[22,23]^ As a new thermal ablation method, microwave ablation (MWA) for NSCLC is likely to be increasingly used, given its theoretical advantages over radiofrequency ablation (RFA) including a less severe heat sink effect and faster, greater heating.^[24]^ We previously reported that advanced and medically inoperable stage I NSCLC could benefit from MWA therapy. Major complications as a result of MWA are rare and tolerable.^[25–28]^ Therefore, we performed the retrospective multicenter study to explore the potential benefit of MWA in treating EGFR acquired resistance NSCLC with extra-CNS oligoprogressive disease.

## 2 Materials and methods

### 2.1 Patients and eligibility

We conducted a retrospective multicenter study at Shandong Provincial Hospital Affiliated to Shandong University, Sun Yat-sen University Cancer Center, Jinan Military General Hospital of Chinese People's Liberation Army and Weifang People's Hospital Affiliated to Weifang Medical University between March 2010 and January 2016. Patients eligible for inclusion in this retrospective analysis included histologically or cytologically proven EGFR-mutant NSCLC treated with erlotinib or gefitinib, objective clinical benefit from erlotinib or gefitinib monotherapy, experienced progression of disease despite the maintenance of erlotinib or gefitinib. EGFR mutations (exon 19 deletions or exon 21 L858R mutations) were examined either through direct sequencing or allele-specific polymerase chain reaction assays. Objective clinical benefit of EGFR-TKIs was defined by either: complete or partial response (CR or PR), or durable stable disease (≥6 months) according to the criteria for acquired resistance proposed by Jackman et al.^[9]^ We collected initial baseline clinical characteristics including sex, age at diagnosis, smoking status, tumor histology, prior therapy, date of diagnosis of any known extra-CNS involvement and sites of metastatic disease of all enrolled patients by retrospective collection from electronic records.

This study was performed in accordance with the declaration of Helsinki and the ethical guidelines and regulations of China. The protocol was approved by the ethics committees of each center before the initiation of enrollment. Written informed consent was obtained from all patients before enrollment in the study.

### 2.2 Treatment and follow-up

All EGFR-mutant patients received erlotinib or gefitinib starting at 150 mg or 250 mg once a day. Patients received routine chest computed tomography (CT) every 1 to 2 months to assess the local response according to the response evaluation criteria in solid tumors.^[29]^ Additional procedures including CT, magnetic resonance imaging, bone scintigraphy and positron emission tomography/CT were applied to evaluate extrapulmonary symptoms and metastatic sites. All patients randomly received MWA or chemotherapy after progression on EGFR-TKIs. In the MWA group, once the acquired resistance was identified patients received MWA followed by the same targeted therapy. After 1 month of MWA, a new baseline for surveillance was established. At 1, 3, 6, 9, and 12 months after MWA, patients received CT scan to assess their response to treatment and to identify adverse events. The patients then underwent imaging every 3 months surveillance thereafter. Sites of first progression were documented. Patients in the chemotherapy group received 2 to 6 cycles of platinum-based systemic chemotherapy followed by the same EGFR-TKI after acquired resistance, and detailed chemotherapy regimens are listed in the Table S1. Chemotherapy was paused until disease progression or unacceptable toxicity.

### 2.3 MWA procedure

Instrumentation: (1) MTC-3C microwave ablation system (Nanjing Qi Ya Research Institute of Microwave Electric, China. Registration standard: YZB/country 1408–2003. NO: SFDA (III) 20073251059) or (2) ECO-2450B microwave ablation system (Nanjing, ECO Microwave Institute, China. Registration standard: YZB/country1475–2013. No: SFDA (III) 20112251456) was used for MWA treatment. Generally, we set the microwave emission frequency at 2450 ± 50 MHz and ablation power between 60 and 80 W. The effective length of the antenna is 100 to 180 mm and 14G to 20G outside diameter. In addition, a water circulation cooling system was applied to reduce the antenna surface temperature. For tumors ≤3.5 cm and 3.6 to 5.0 cm, we used single or double antennae, respectively.^[25,26,30]^ The whole procedures of MWA were performed under CT guidance and the detailed procedures were described in our previous publications.^[25,26]^ All patients received a CT scan immediately after MWA to monitor the shape and size of the lesions, as well as to determine any potential complications including pneumothorax, bleeding or others. The proposed ablative margin was 0.5 cm.

### 2.4 Statistical analysis

Pearson's Chi-square (or Fisher's exact) and *t*-test were used to determined differences between groups. Median progression-free survival (PFS1) was calculated from time of initiation of targeted therapy to first progression of disease or clinical progression (as assessed by clinician). PFS2 was defined from the time of first progression to second progression after MWA or chemotherapy. OS was calculated from the time of diagnosis to the date of last follow-up or death from any case. Survival curves were estimated using Kaplan–Meier method. Patients who did not experience progression or death during the study time were censored at the time of the last available follow-up. Univariable and multivariable Cox proportional hazards regression models were used to assess the association between each of the variables and PFS2 or OS. For the multivariable analysis model, we include treatment strategy variable, which was statistically significant on univariable analysis.

We used bootstrapping for bias correction (n = 1000 bootstrap samples). Random samples (sample size was equivalent to original cohort) were drawn from the original data set with replacement. Then, we applied the same Cox model, which was derived from the original cohort to the bootstrap samples. One thousand bootstrapping-based model performance indices were independently generated by repeating this process 1000 times. The mean value of the 1000 performance indices is the bias-corrected estimate, which is considered to estimate the nomogram performance that could be expected in a separate but similar patient population. The average and standard deviation of these 1000 bootstrap samples of the regression parameter were calculated as the bootstrap estimate and standard error (SE) of the parameter. Statistical analysis was performed using SPSS for Windows Version 17.0 (IBM, Chicago, IL) and R software version 3.1.1 (The R Foundation for Statistical Computing). All analyses were two sided with a *P*-value <0.05 considered statistically significant.

## 3 Results

### 3.1 Patients characteristics

From Jan 2010 to Jan 2016, 54 patients were enrolled in this study. Twenty-eight patients received MWA while 26 patients received chemotherapy after acquired resistance to TKIs. The characteristics of all patients are shown in Table 1. EGFR mutations were confirmed in all patients, with exon 19 deletion mutation being most common (50% in MWA group, 46.2% in chemotherapy group), followed by exon 21 L858R mutation (39.3% in MWA group, 38.5% in chemotherapy group) and other type of mutation (10.7% in MWA group, 15.3% in chemotherapy group). All patients had oligoprogressive disease (<5 sites of disease) at the time of PFS1 and the pattern of progression for these patients is also summarized in Table 1. There was no significant difference in age, sex, smoking history, stage at diagnosis, best response to TKI, and sites of metastatic disease between the MWA group and chemotherapy group.

**Table 1:**
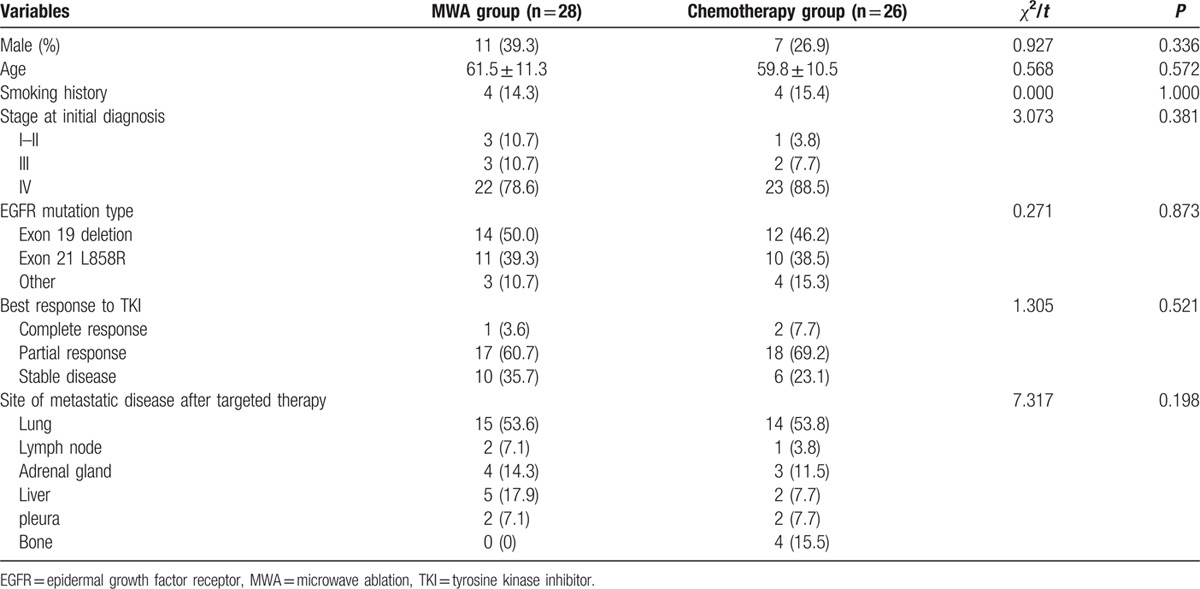
Patients and tumor characteristics.

### 3.2 Treatment procedures and complications after MWA

All patients with extra-CNS oligoprogressive disease received MWA followed by continuation of targeted therapy in the present study. The median time from the first proression to MWA was 3 weeks. Thirty-one metastatic sites in 28 cases were ablated. The mean size of the metastatic sites was 3.1 cm (range, 1–6.4 cm). Most MWAs were well tolerated. No patient died during the procedure or within 30 days after MWA. Pain was the common side effect and 10 patients (35.7%) suffered moderate pain after MWA, but no severe post-ablation pain occurred. Post-ablation syndrome including fever (under 38.5 °C), fatigue, general malaise, nausea, and vomiting, etc. occurred in 9 patients. Postoperative pneumothorax was observed in 10 patients (35.7%), in which 2 of them received chest tube drainage. Five patients (17.8%) had pleural effusion (of which 1 case underwent chest tube insertion) after the MWA. Hemoptysis occurred in 2 cases (7.1%) and the conventional application of hemostatic agents including snake venom thrombin, glucocorticoids, could effectively stop bleeding. The average length of hospital stay was 4.3 days (3–17 days).

### 3.3 Outcomes

**Figure 1:**
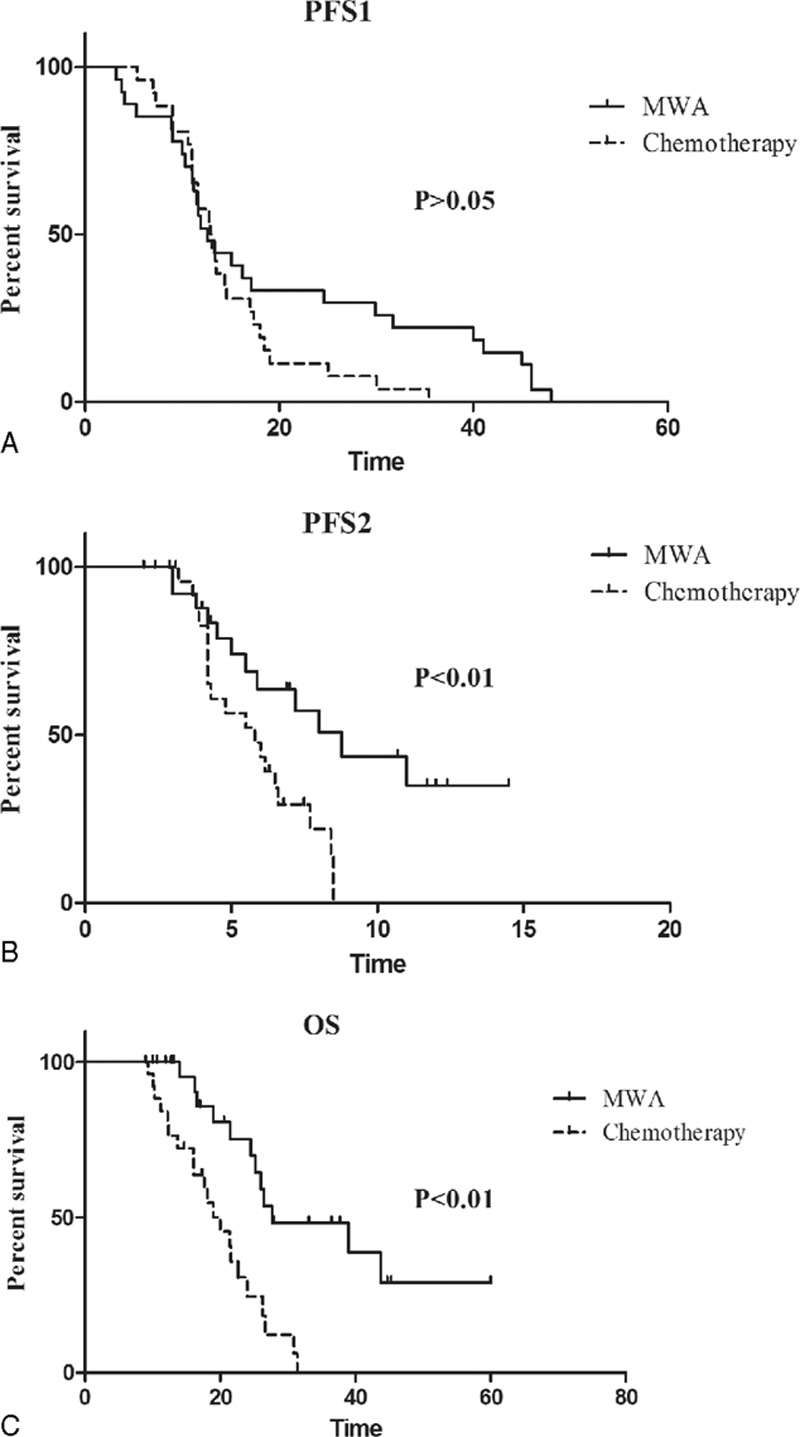
Kaplan–Meier survival curves of PFS1, PFS2, and OS for MWA group patients (solid lines) and chemotherapy group (dashed). (A) Median PFS1 was 12.6 months for MWA group and 12.9 months for chemotherapy group (HR 0.63, CI 0.350–1.142, *P* = 0.13). (B) Median PFS2 was 8.8 months for MWA group and 5.8 months for chemotherapy group (HR 0.357, CI 0.169–0.751, *P* = 0.0067). (C) Median OS was 27.7 months for MWA group and 20.0 months for chemotherapy group (HR 0.238, CI 0.112–0.505, *P* = 0.0002). CI = confidence interval, HR = hazard ratio, MWA = microwave ablation, OS = overall survival, PFS1 = progression-free survival.

**Figure 2:**
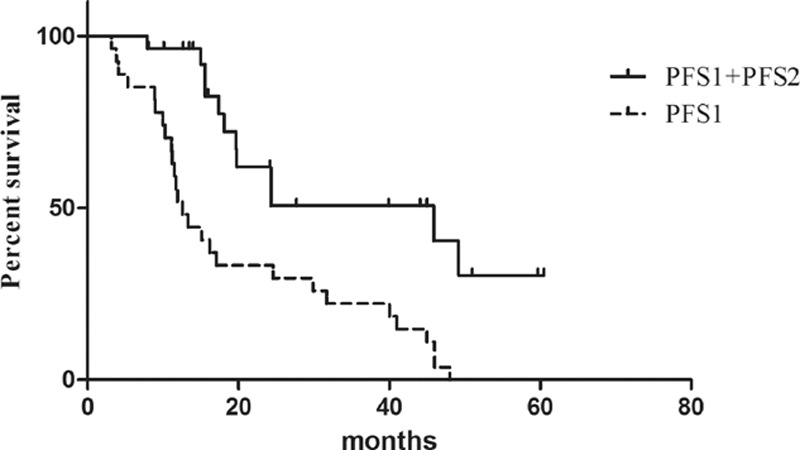
Kaplan–Meier survival curves of PFS1 and PFS1 + PFS2 of all 28 patients treated with MWA. MWA = microwave ablation, PFS1 = progression-free survival.

The median follow-up for this study was 23.5 months (range, 7–70 months). Among the 28 patients in the MWA group, 11 of them progressed after MWA during the study period, and 7 patients died. While 21 of 26 patients in the chemotherapy group died. The difference of PFS1 in both groups was not significant (12.6 months vs. 12.9 months, HR 0.63, CI 0.350–1.142, *P* = 0.13) (Fig. 1A). However, the patients in MWA group had a significantly longer PFS2 than those in chemotherapy group (8.8 months vs. 5.8 months, HR 0.357, CI 0.169–0.751, *P* = 0.007) (Figs. 1B and 2). In addition, the OS in MWA group was also longer than that in chemotherapy group (27.7 months vs. 20.0 months, HR 0.238, CI 0.112–0.505, *P* = 0.0002) (Fig. 1C).

The variables listed in Table 1 were examined (Cox Regression) for their association with PFS2 and OS. Results of the univariate analyses are shown in Table 2. Treatment strategy with univariate significance (*P* < 0.05) was selected for the multivariate analysis, and the detailed results and the hazard ratios of the multivariate analysis estimated by Cox regression are listed in Table 2. We confirmed the results of the multivariate analysis by using the bootstrap resampling technique mentioned above. We observed the closeness of the bootstrap estimates to those obtained in the final Cox model, which validated the final results (Table 3).

**Table 2:**
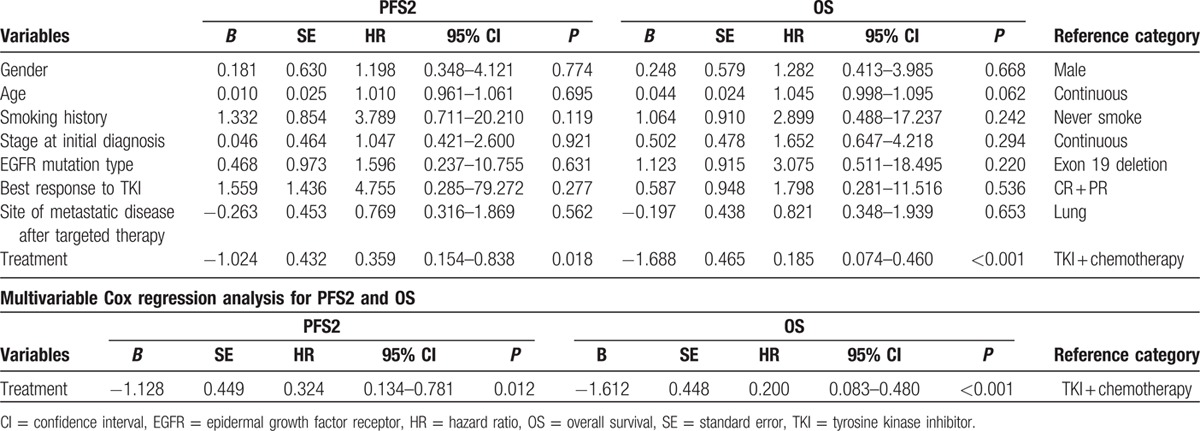
Results of Cox regression analyses.

**Table 3:**
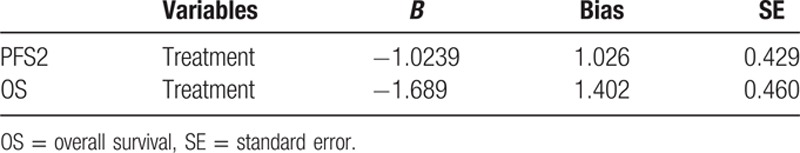
Summary of variables for 1000 bootstrap samples.

**Figure 3:**
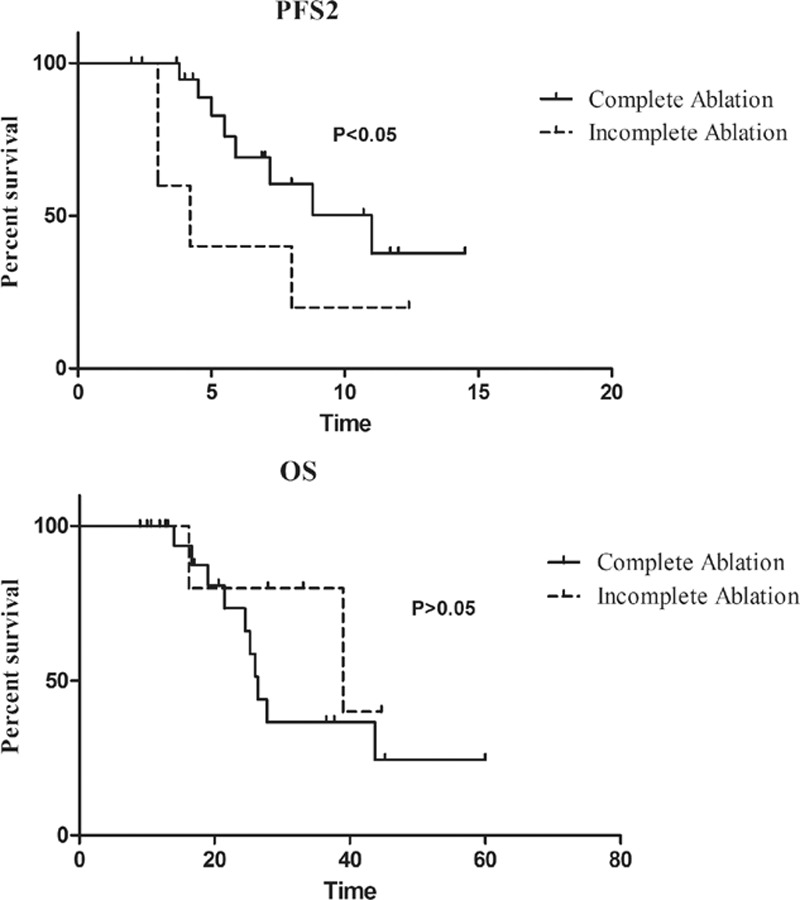
Kaplan–Meier survival curves of PFS2 and OS between complete ablation and incomplete ablation. The median PFS2 was 11 months for complete ablation and 4.2 months for incomplete ablation (HR 0.287, CI 0.062–1.339, *P* = 0.02). The difference of OS between the two groups was not significant (39 months vs. 26.4 months, HR 1.876, CI 0.536–6.57, *P* > 0.05). CI = confidence interval, HR = hazard ratio, OS = overall survival.

In the MWA group, the overall complete ablation rate achieved 78.6% (22 of 26). The median PFS2 for complete ablation was significantly longer than that for incomplete ablation (11 vs. 4.2 months, hazard ratio: 0.2874, 95% CI of the ratio: 0.062–1.339, *P* < 0.05). The difference of OS between the two groups was not significant (39 vs. 26.4, *P* > 0.05) (Fig. 3).

## 4 Discussion

The past decade witnessed the dramatic alteration in the treatment of NSCLC with the identification of somatic gene mutations that underlie tumor initiation and maintenance. EGFR mutations were first identified in lung cancer after clinical benefit to EGFR tyrosine kinase inhibitors was observed. First-line TKI therapy is recommended in NSCLC harboring an EGFR mutation. Unfortunately, most patients who initially benefit from erlotinib or gefitinib ultimately developed acquired resistance to these drugs, and there are limited options for these patients. Patients treated with erlotinib or gefitinib monotherapy in this study had a median PFS1 of 12.6 months, which is similar with that of chemotherapy group (12.9 months) and consistent with literature precedent, especially with the data of East Asian patients,^[3,5,7,31]^ indicating that we do not seem to have preselected a more indolent population for MWA within this study.

Cytotoxic chemotherapy or participation in a clinical trial was considered as the standard treatment in this setting. EGFR-TKI was replaced with docetaxel or pemetrexed but efficacies of these agents were not satisfactory. The median PFS2 of pemetrexed and docetaxel was 3.9 months and 4.1 months, respectively.^[32,33]^ Pemetrexed in combination with erlotinib or gefitinib after disease progression showed better response and was associated a median PFS2 of 7.0 months.^[19]^ Recent data suggest that for patients with EGFR-mutation who develop highly localized disease progression (oligoprogressive disease), local therapy to these sites with surgery or radiation, in combination with ongoing use of the same TKI, might also be clinically beneficial. It was recently reported that Oncogene-addicted NSCLC with CNS and/or extra-CNS oligoprogressive disease on relevant targeted therapies is suitable for local ablative therapy and continuation of the targeted agents, and is associated with more than 6 months of additional disease control.^[22]^ Moreover, Yu et al^[23]^ reported EGFR-mutant lung cancers with acquired resistance to EGFR-TKI therapy are amenable to local therapy including surgery or radiation followed by continued targeted therapy, and 10 months additional disease control was observed. However, there is little published data about the use of MWA for oligoprogressive disease on therapy. This is the first study to evaluate the efficacy and safety of MWA as a local therapy for patients with TKI acquired resistance. In this study, the MWA group had a similar PFS1 with that of chemotherapy group (control group). However, the PFS2 and OS were significantly longer in patients of MWA group compared with chemotherapy group. Our data suggest that MWA in combination with continuation of the TKIs may be associated with longer PFS and OS in patients with EGFR-mutation on targeted therapy who developed less than two systemic progressive lesions. These results expand on the role of local therapy in EGFR-mutant NSCLC that developed extra-CNS oligoprogressive disease during TKI treatment and suggest that these patients may benefit from the MWA.

The mechanism of acquired resistance to EGFR-TKIs in NSCLC is still not well understood. Several molecular mechanisms have been elucidated and generally can be classified into 3 main categories: alterations in EGFR itself, activation of bypass signaling pathways, and phenotypic transformation.^[34]^ The most frequent mechanism of acquired resistance to EGFR-TKIs is the secondary T790M mutation in EGFR which is identified in approximately 50 to 60% of patients. However, in metastatic cancers, biological heterogeneity among tumour cells pre-exists at the time of clinical presentation and tumors resistant to EGFR-TKIs may be composed of a heterogeneous mix of TKI-sensitive and TKI-resistant cells. Continuation of targeted therapy can lead to ongoing benefit in nonprogressing clones, which have not developed acquired resistance. Our data indicated that the efficacy of MWA together with continuation of TKIs may be superior to TKIs treatment alone, suggesting local microwave ablative therapy of the resistant clone before widespread dissemination is associated with prolonged disease control.

In the present study, the most common complication for MWA therapy was pneumothorax, followed by pleural effusion and hemoptysis. Complications can be controlled by proper treatments and there is no patient died during or within 30 days after the procedure. Therefore, MWA is a safe and well tolerable strategy for NSCLC patients.

Certainly, our study does have some limitations. The main limitation of this study lies in its retrospective nature and relatively small number of treated patients. Further limitations include the absence of pathological proof in metastatic diseases and identification of the acquired resistance molecular characteristics. Prospective multicenter evaluation of MWA with large sample size is needed to define more clearly the effectiveness and safety of this treatment strategy.

Despite of these limitations, our results show that MWA with continued EGFR inhibition may extend disease control by over 8 months in patients with oligoprogressive disease. MWA as a local therapy for oligoprogressive disease should be considered for NSCLC with acquired resistance to EGFR-TKIs.

## Supplementary Material

Supplemental Digital Content
