# Supplementary material for: Local microwave ablation with continued EGFR tyrosine kinase inhibitor as a treatment strategy in advanced non-small cell lung cancers that developed extra-central nervous system oligoprogressive disease during EGFR tyrosine kinase inhibitor treatment: A pilot study
Source: Medicine (Baltimore). 2016 Jun 24;95(25):e3998. doi: 10.1097/MD.0000000000003998 (PMC4998341; doi:10.1097/MD.0000000000003998)
Supplement: Supplemental Digital Content [file medi-95-e3998-s001.doc]

| Patient | Gender | Age | Chemotherapy regimen | Cycles | Response to chemotherapy |
| --- | --- | --- | --- | --- | --- |
| 1 | M | 64 | Docetaxel + cisplatinum | 4 | SD |
| 2 | F | 68 | Pemetrexed + cisplatinum | 3 | PD |
| 3 | F | 38 | Pemetrexed + cisplatinum | 4 | SD |
| 4 | F | 42 | Irinotecan + paclitaxel | 3 | PD |
| 5 | F | 60 | Pemetrexed + cisplatinum | 3 | SD |
| 6 | M | 63 | Docetaxe + cisplatinum | 3 | PD |
| 7 | F | 63 | Pemetrexed + cisplatinum | 4 | PR |
| 8 | F | 69 | Docetaxel + cisplatinum | 3 | PD |
| 9 | F | 42 | Pemetrexed + cisplatinum | 6 | PR |
| 10 | F | 39 | Pemetrexed + cisplatinum | 2 | SD |
| 11 | F | 66 | Pemetrexed + cisplatinum | 5 | PR |
| 12 | F | 51 | Docetaxel + cisplatinum | 2 | PD |
| 13 | F | 60 | Pemetrexed + cisplatinum | 3 | SD |
| 14 | M | 53 | Docetaxel + cisplatinum | 4 | PD |
| 15 | M | 62 | Pemetrexed + cisplatinum | 2 | PR |
| 16 | M | 55 | Pemetrexed + cisplatinum | 4 | SD |
| 17 | F | 61 | Pemetrexed + carboplatin | 3 | PD |
| 18 | F | 79 | Pemetrexed + cisplatinum | 4 | PR |
| 19 | F | 66 | Docetaxel + cisplatinum | 5 | PR |
| 20 | M | 74 | Pemetrexed + cisplatinum | 3 | PR |
| 21 | F | 56 | Docetaxel + cisplatinum | 4 | SD |
| 22 | F | 71 | Docetaxel + cisplatinum | 3 | PD |
| 23 | M | 68 | Pemetrexed + cisplatinum | 3 | SD |
| 24 | F | 59 | Pemetrexed + cisplatinum | 4 | SD |
| 25 | F | 62 | Docetaxel + cisplatinum | 2 | PD |
| 26 | F | 64 | Pemetrexed + cisplatinum | 3 | SD |

Table S1 Chemotherapy regimen of each patient in the chemotherapy group
